# Valence can control the nonexponential viscoelastic relaxation of multivalent reversible gels

**DOI:** 10.1126/sciadv.adl5056

**Published:** 2024-05-15

**Authors:** Hugo Le Roy, Jake Song, David Lundberg, Aleksandr V. Zhukhovitskiy, Jeremiah A. Johnson, Gareth H. McKinley, Niels Holten-Andersen, Martin Lenz

**Affiliations:** ^1^ Université Paris-Saclay, CNRS, LPTMS, 91405, Orsay, France.; ^2^ Institute of Physics, École Polytechnique Fédérale de Lausanne (EPFL), 1015 Lausanne, Switzerland.; ^3^ Department of Materials Science and Engineering, Massachusetts Institute of Technology, 77 Massachusetts Avenue, Cambridge, MA 02139, USA.; ^4^ Department of Mechanical Engineering, Stanford University, Stanford, CA 94305, USA.; ^5^ Department of Chemical Engineering, Massachusetts Institute of Technology, 77 Massachusetts Avenue, Cambridge, MA 02139, USA.; ^6^ Department of Chemistry, Massachusetts Institute of Technology, 77 Massachusetts Avenue, Cambridge, MA 02139, USA.; ^7^ Department of Chemistry, University of North Carolina at Chapel Hill, Chapel Hill, NC 27599, USA.; ^8^ Department of Mechanical Engineering, Massachusetts Institute of Technology, 77 Massachusetts Avenue, Cambridge, MA 02139, USA.; ^9^ Department of Bioengineering and Materials Science and Engineering, Lehigh University, Bethlehem, PA 18015, USA.; ^10^ PMMH, CNRS, ESPCI Paris, PSL University, Sorbonne Université, Université de Paris, F-75005 Paris, France.

## Abstract

Gels made of telechelic polymers connected by reversible cross-linkers are a versatile design platform for biocompatible viscoelastic materials. Their linear response to a step strain displays a fast, near-exponential relaxation when using low-valence cross-linkers, while larger supramolecular cross-linkers bring about much slower dynamics involving a wide distribution of timescales whose physical origin is still debated. Here, we propose a model where the relaxation of polymer gels in the dilute regime originates from elementary events in which the bonds connecting two neighboring cross-linkers all disconnect. Larger cross-linkers allow for a greater average number of bonds connecting them but also generate more heterogeneity. We characterize the resulting distribution of relaxation timescales analytically and accurately reproduce stress relaxation measurements on metal-coordinated hydrogels with a variety of cross-linker sizes including ions, metal-organic cages, and nanoparticles. Our approach is simple enough to be extended to any cross-linker size and could thus be harnessed for the rational design of complex viscoelastic materials.

## Teaser

Cross-linker valency impacts the mechanical relaxation of reversible gels.

## INTRODUCTION

Soft hydrogels are ubiquitous in biology and dictate the mechanics of cells and tissues (*1*). Because of their biocompatibility, synthetic hydrogels are thus prime candidates to serve as robust soft tissue implants, although fine control of their viscoelastic properties is crucial for their success in this role (*2*, *3*). In simple viscoelastic materials, stress relaxes according to a single exponential with a single relaxation time. This is not however the case for most biological materials such as cells (*4*), tissues (*5*), mucus (*6*), and biofilms (*7*). Instead, their relaxation is characterized by a broad distribution of relaxation times (*8*). Such relaxation is often heuristically described by a stretched exponential

$$\sigma \left(t\right)\propto e^{-{\left(t/\tau \right)}^{\alpha }}$$ (1)

where smaller values of the exponent α ∈ ]0; 1[ denote broader distributions of relaxation timescales (*9*). Other similarly phenomenological fitting functions include power-law dependences of σ on *t* (*10*–*12*) and log-normal distributions of the relaxation times (*13*, *14*).

**Fig. 1.**
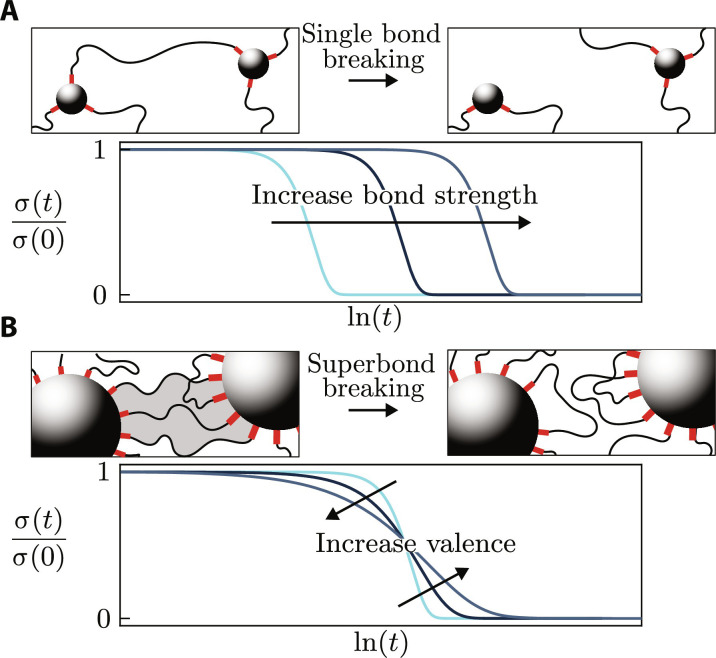
High-valence cross-linkers yield a slow, potentially complex unbinding dynamics. (**A**) Hydrogels held together by small cross-linkers relax over the timescale associated with the unbinding of a single polymer strand. There, stress decays exponentially in response to a step strain: σ(*t*)/σ(0) = exp(−*t*/τ). Since *t*/τ = exp(ln *t* − ln τ), changes in the timescale τ shift the σ versus *ln t* relaxation curve horizontally but do not alter its shape. (**B**) In contrast, relaxation events in the presence of high-valence cross-linkers require the simultaneous unbinding of many polymer stands. The associated timescale is long and highly variable depending on the number of strands involved in the superbond (gray shade). As a result, the stress relaxation of such gels is no longer exponential, and the precise shape of the relaxation curve strongly depends on the valence of the cross-linkers. In practical cases, changes in valence are typically accompanied by an additional shift of the curves toward larger relaxation times not shown in this illustrative schematic.

Associative gels, which relax by a succession of binding and rebinding events (*15*), offer a promising route to design controllable viscoelastic materials. It is thus possible to tune their relaxation time by modifying the dissociation rate (*16*) of their cross-linkers (*17*, *18*). Although this chemistry-based approach allows tuning of the overall stress relaxation time as illustrated in Fig. 1A, less is known about the different approaches to tune the shape of the stress relaxation curve of reversible hydrogels. Accordingly, most existing models for the relaxation of multivalent gels focus on regimes dominated by a single relaxation timescale (*19*), leading to exponential relaxation (*20*). Control over the distribution of relaxation timescales could however be achieved in synthetic hydrogels connected with multivalent dynamic cross-linkers such as nanoparticles (NPs) (*21*), metal-organic cages (*22*), clay (*23*), and latex beads (*24*), which are known to exhibit nonexponential viscoelastic relaxation. Here, we aim to elucidate this emergence of a wide distribution of timescales in materials with high-valence cross-linkers to enable the rational design of complex gels (Fig. 1B). Here, we use the term “valence” to designate the number of polymer strands that a cross-linker can bind, a property sometimes also referred to as their “functionality” (*25*).

We propose that the emergence of a broad distribution of relaxation timescales arises from microscopic events consisting of the severing of the physical connection between two cross-linkers. We first propose a model where this connection, hereafter termed “superbond,” breaks if all its constitutive cross-linkers are detached at the same time (Fig. 1B). We show that the breaking time of a superbond increases exponentially with the number of strands involved, consistent with previous observation (*26*). As a result of this strong dependence, small spatial heterogeneities in the polymer concentration may result in widely different relaxation times from one superbond to the next. Such exponential amplification of relaxation times originating from small structural differences forms the basis of models previously used to describe the relaxation of soft glasses (*27*–*29*). In contrast with these studies, our approach explicitly models the microscopic basis of this amplification. That allows it to not only recover relaxation curves virtually indistinguishable from those discussed in previous studies but to also predict the influence of temperature and cross-linker valence on the macroscopic stress relaxation observed in the resulting gel. The details of the polymer strand morphology are not central to this influence, and we thus enclose them in a few effective parameters that could be derived from first principles in specialized models related to specific implementations of our basic mechanism. To confirm these predictions, we conduct experiments on hydrogels with four distinct cross-linker types of different sizes and find that our model quantitatively reproduces multiple relaxation curves using this small set of microscopic parameters. Last, we show that several phenomenological fitting functions used in the literature can be recovered as asymptotic regimes of our analytical model.

## RESULTS

Model of a single superbond

We first model a single superbond in the simple, experimentally relevant (*22*) case of strong interactions combined with short polymers, which implies negligible entanglements as discussed in Methods. In the limit of very large and rigid cross-linkers, the polymer layer around a cross-linker is locally planar, its structure is not affected by small fluctuations of polymer concentration, and its thickness fixes the distance between cross-linkers (*22*, *30*). We thus use the simplifying assumption that all individual bonds participating in a superbond are identical and noninteracting, an approximation whose validity we ultimately assess through comparisons with experiments.

**Fig. 2.**
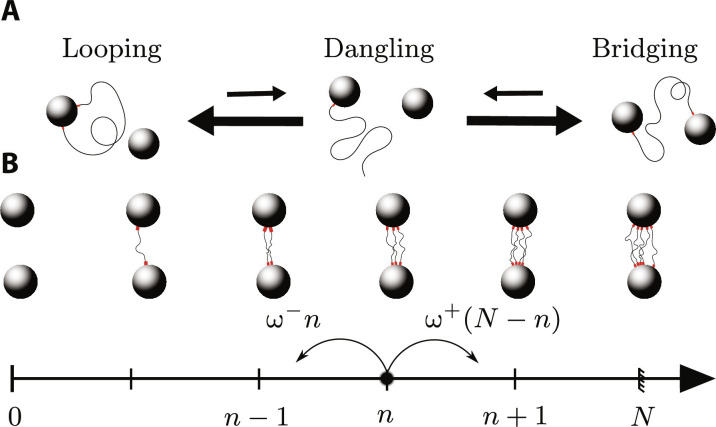
We model superbond breaking as the disconnection of many independent polymer strands. (**A**) Disconnecting a single polymer strand requires going through a high-energy, short-lived dangling state (larger arrows indicate faster transitions). The looping and bridging states both have two polymer–cross-linker bonds and therefore have the same energy. (**B**) Individual strands in a superbond attach and detach independently, resulting in a one-dimensional random walk in the number *n* of attached strands (Eq. 3). Here, we only draw the bridging strands and not the looping strands.

We model the attachment and detachment of a single polymer strand from a pair of cross-linkers as shown in Fig. 2A. When both its ends are bound, the strand may or may not connect two different cross-linkers. The corresponding “bridging” and “looping” states have the same energy since we assume the polymer strand to be completely flexible, and we denote by Δ*S* the entropy difference between them. To transition between these two states, the strand must disconnect one of its ends and form a “dangling” state. The disconnection of the strand in this state implies an energy barrier Δ*E* that is much larger than the thermal energy *k_B_T* = β^−1^. This implies that the dangling state is short-lived, and thus need not be explicitly included in our modeling. Our approximation scheme implies that the overall rate ω^+^ and ω^−^ to go from the looping to the bridging state and back are constant. They read

$$\omega^{+}=\frac{1}{\tau_{0}}e^{-\beta \Delta E}\;\omega^{-}=\frac{1}{\tau_{0}}e^{-\beta \Delta E+\Delta S}$$ (2)

where the typical timescale τ_0_ takes into account the entropy difference between the looping and dangling state. At equilibrium, we denote the probability for a single polymer strand to create a bridge as *p*_on_ = 1 − *p*_off_ = 1/(1 + *e^ΔS^*).

We now consider the dynamic of a single superbond. In the limit of small polymer strands and large cross-links, we neglect the exchange of polymer strands between superbonds. Each superbond thus fluctuates independently of the other superbonds belonging to the same cross-linker. In that case, the total number of polymer strands in a superbond is fixed in time, and we denote it by *N*. Within our approximation of independent attachment and detachment of the individual polymer strands, the superbond undergoes the Markov process illustrated in Fig. 2B and the probability *P_n_*(*t*) for *n* strands to create bridges between the two cross-linkers at time *t* satisfies the master equation

∂tPn(t)=(N−n+1) ω+Pn−1(t)+(n+1) ω−Pn+1(t)−[(N−n) ω++n ω−]Pn(t) (3)

which can be derived as the master equation whereby a state with *n* bridging and (*N* − *n*) looping strands gains one with transition rate (*N* − *n*)ω^+^ and loses one with *n*ω^−^ (*31*). Equation 3 ensures that the number of bridging strands *n* is always comprised between 0 and *N*, which must be the case as each strand in our model has exactly two bonds and is thus either bridging or looping (Fig. 2).

To determine the rate at which a superbond breaks, we set an absorbing boundary condition *P*_0_(*t*) = 0 and define its survival probability as *S*(*t*) = ∑_*n*^N^ = 1_* P_n_*(*t*). In the limit *N* ≫ 1 where a large number of strands are involved in the superbond, we show in section S1 that the detachment of the two beads is analogous to a Kramers escape problem. We thus prove that the survival probability decays as a single exponential *S*(*t*) = exp(−*t*/τ*_N_*) (*32*) with an average detachment time

$$\tau_{N}\underset{\;N\to \infty }{∼}\frac{\tau_{0}e^{\beta \Delta E}}{{\mathrm{Np}}_{\text{off}}^{N}}$$ (4)

The breaking of the superbond can thus be assimilated to a Poisson process with rate 1/τ_N_ regardless of the initial condition P_n_(0). The strong, exponential dependence of τ_N_ on N implies that any dispersity in the number of strands involved in a superbond may result in a wide distribution of timescales.

### Model of the relaxation of a gel

Two factors influence the dispersity of *N*. First, its value is constrained by the available space at the surface of each cross-linker, which we model by setting an upper bound *N*_sat_ on the number of polymer strands (in a loop or a bridge) participating in any superbond. Second, depending on the local density of polymer in the vicinity of the superbond, the actual number of strands present may be lower than *N*_sat_. In the regime where the polymer solution surrounding the cross-linkers is dilute, polymer strands are independently distributed throughout the system. As a result, the distribution of local strand concentrations within a small volume surrounding a superbond follows a Poisson distribution. We thus assume that *N* is also described by a Poisson distribution up to its saturation at *N*_sat_

$$p\left(N\right)=\left\{ \begin{array}{cc} \frac{{\bar{N}}^{K}e^{-\bar{N}}}{N!} & \text{for}\;N<N_{\text{sat}}\\ \sum_{K=N_{\mathrm{sat}}}^{+\infty }\frac{{\bar{N}}^{K}e^{-\bar{N}}}{N!} & \text{for}\;N=N_{\text{sat}} \end{array}\right.$$ (5)

where $\bar{N}$ would be the average number of strands in a superbond in the absence of saturation and thus depends on the ratio of polymer to cross-linker concentration. Note that the specific form of the distribution used in Eq. 5 does not substantially modify our results, as discussed later.

In response to a step strain, we assume that each superbond is stretched by an equal amount and resists the deformation with an equal force before breaking. Superbonds may subsequently reform, but the newly formed bonds are not preferentially stretched in the direction of the step strain and therefore do not contribute to the macroscopic stress on average. Denoting by *t* = 0 the time at which the step strain is applied and by σ(*t*) the resulting time-dependent shear stress, the progressive breaking of the initial superbonds results in the following stress response function

$$\frac{\sigma \left(t\right)}{\sigma \left(t=0\right)}=\sum_{N=1}^{N_{\text{sat}}}\;\frac{p\left(N\right)}{1-p\left(0\right)}e^{-t/\tau_{N}}$$ (6)

**Fig. 3.**
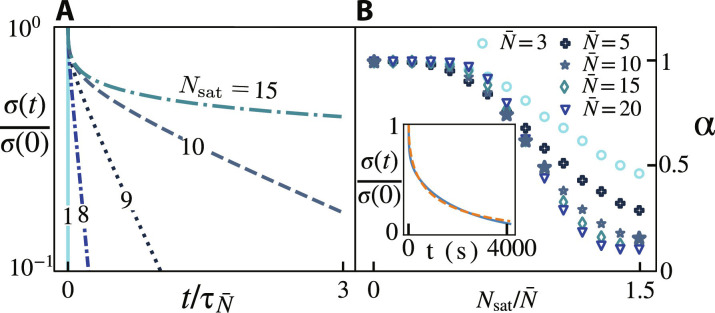
Influence of the valency on the stretch exponent. (**A**) Disperse, high-valence superbonds initially display a nonexponential mechanical relaxation and then cross over to an exponential regime when only the saturated superbonds remain. Curves plotted from Eq. 6 with *p*_off_ = 0.2, $\bar{N}=10$ and different values of *N*_sat_ as indicated on each curve. (**B**) Relationship between the stretch exponent α quantifying the nonexponential character of the relaxation and the microscopic parameter $N_{\text{sat}}/\bar{N}$ . Here *p*_off_ = 0.2. A low $N_{\text{sat}}/\bar{N}$ gives an exponential relaxation (α ≃ 1), while a larger $N_{\text{sat}}/\bar{N}$ leads to a more complex behavior (α < 1). While α appears to converge to a finite value for large $N_{\text{sat}}/\bar{N}$ for the largest values of $\bar{N}$ , this behavior is contingent on our choice of fitting interval. This issue does not affect the rest of the curves. Large stars correspond to the curves represented in (A). Inset, illustration of the quality of the fits between the heuristic stretched exponential (dashed orange line, Eq. 1) and our prediction (solid blue line, Eq. 6).

While the breaking times τ*_N_* are unaffected by the applied stress in the linear response regime, nonlinearities could easily be included in our formalism by making Δ*S* stress-dependent and thus favoring strand detachment. The relaxation described in Eq. 6 occurs in two stages. At long times *t* ≫ τ_*N*_sat__, few short-lived superbonds remain. Saturated superbonds (*N* = *N*_sat_) dominate the response, and Eq. 6 is dominated by the last term of its sum. As a result, the stress relaxes exponentially over time, as seen from the linearity of the log-lin curves of Fig. 3A for large values of *t*. Systems with smaller values of *N*_sat_ manifest this regime at earlier times; in the most extreme case, where superbonds involve at most a single polymer strand (N_sat_ = 1), the relaxation of the system is fully exponential and extremely fast as compared to systems with higher *N*_sat_. Over short times (*t* ≪ τ_*N*_sat__), stress relaxation involves multiple timescales. This nonexponential regime is apparent on the left of Fig. 3A. These two regimes have already been reported in several experimental gels connected by multivalent dynamic cross-linkers (*1*, *30*).

While Eq. 6 is not identical to the stretched exponential of Eq. 1, the inset of Fig. 3B shows that they are remarkably close in practice. We thus relate the stretch exponent α to the saturation number *N*_sat_ by fitting a stretched exponential to our predicted stress response function over the time interval required to relax 90% of the initial stress (Fig. 3B). The fits are very close matches and consistently give correlation factors *r*^2^ > 0.98 (see detailed plots in fig. S2). If $N_{\text{sat}}≲0.5\bar{N}$ then α ≃ 1, indicating a nearly exponential relaxation. In that case, superbond saturation occurs well before the peak of the Poisson distribution of *N*. Physically, this implies that the local polymer concentration surrounding most superbonds is sufficient to saturate them. As almost all superbonds are saturated, they decay over the same timescale τ_*N*_sat__. As a result, the material as a whole displays an exponential relaxation. For larger values of *N*_sat_, the Poisson distribution is less affected by the saturation, and the dynamics is set by the successive decay of superbonds involving an increasing number of strands, implying lower values of α. The larger the value of $\bar{N}$, the sharper the crossover between these two regimes.

### Experiments

To validate our model of the effect of cross-linker valence on hydrogel relaxation, we perform step-strain experiments of poly(ethylene glycol) (PEG)–based gels involving a range of cross-link valences and compare Eq. 6 to the resulting relaxation curves. We implement two sets of metal-cooordination chemistry: nitrocatechol-Fe^3+^ coordination and bispyridine-Pd^2+^ coordination. The first set of gels is made with nitrocatechol-functionalized PEG cross-linked by single Fe^3+^ ions with an estimated valence of 3, and by iron oxide NPs. The NPs have a mean diameter of 7 nm with a surface area that allows a valence of ~100 ligands (*21*). The second set of gels are made with bispyridine-functionalized PEG, wherein bis-*meta*-pyridine ligands induce self-assembly of gels that are cross-linked by Pd_2_L_4_ nanocages with a valence of 4, and bis-*para*-pyridine ligands induce self-assembly of gels that are cross-linked by Pd_12_L_24_ nanocages with a valence of 24 (*33*). As shown in Fig. 3, these four distinct gel designs result in a broad range of relaxation behaviors. Overall, large-valence gels and lower temperatures result in longer relaxation times, consistent with the illustration of Fig. 1A. The relaxation curves associated to high-valence cross-linkers are also less steep, consistent with the involvement of a broader distributions of relaxation times and the schematic of Fig. 1B.

To demonstrate the application of the model based on these valency values, we estimate the value of *N*_sat_ associated with each system based on an initial hypothesis that each cross-linker is connected to six nearest neighbors, i.e*.*, *N*_sat_ = valence/6 (rounded to an integer in Table 1). This represents an upper bound estimate (*34*, *35*) of the number of nearest neighbors in the gel; the actual number could be estimated through molecular simulations (*36*).

**Table 1.** Estimated and fitted parameters involved in the comparison between experiment and theory in Fig. 4. The energies are given in units of *k_B_T* for *T* = 300 K. Instead of displaying the parameter τ_0_, we present the more easily interpreted unbinding time of a single polymer strand at 300 K, namely $\tau_{1}=\tau_{0}e^{\beta 300\Delta E}/p_{\text{off}}$.

**Cross-linker**	Fe^3+^	Pd_2_L_4_	Pd_12_L_24_	Nanoparticles
**Estimated valence**	3	4	24	100
*** N***_sat_	1	4	7	17
Δ*** E*** (units of *k_B_T*)	28	24	24	24
*** p***_**off**_	0.08	0.15	0.15	0.36
τ_1_ at *T* = 300 K (s)	1.7	6.1	1.9	0.1
$\bar{N}$	1	6	9	14

At a more detailed level, our assumption that the dynamics of single polymer strand proceeds independently of its environment implies the existence of a single energy scale Δ*E*. As a result, we predict that all timescales involved in the relaxation are proportional to exp(−βΔ*E*). We confirm this through a time-temperature collapse shown in the insets of Fig. 3 (see section S4 for details). This collapse provides us with the value of Δ*E* for each of our four systems, which we report in Table 1. The binding energy value Δ*E* for the Pd_2_L_4_ and Pd_12_L_24_ gels match, as expected from the fact that they originate from the same composition. On the other hand, the Δ*E* of the NP gels appear to be lower than the Fe^3+^ ion gels, which is due to the reduced electron availability of the Fe sites on the NP surface. Moreover, the environment used to make NP gels is more acidic than the ion gels, which modifies the ligand affinity.

**Fig. 4.**
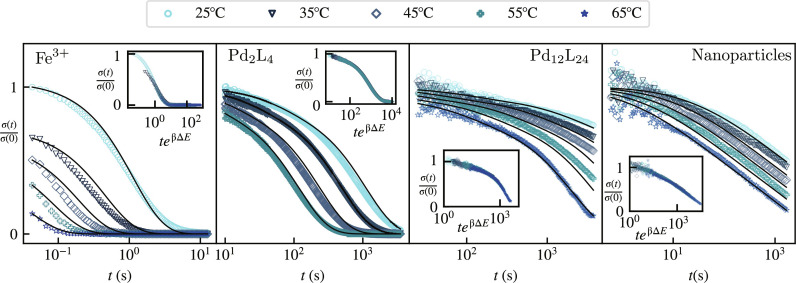
Stress relaxation function for four experimental systems with increasing cross-linker valences (see Table 1 for values). The averaged correlation coefficients for the fits of each panel from left to right are: 0.98, 0.99, 0.94, and 0.96. Here, we use a log-lin scale (unlike in Fig. 3A) to facilitate the visualization a large range of timescales. Alternate representations are available as fig. S5. Symbols are experimental data points, and the lines are the associated fitting curves. Insets, time-temperature collapsed data obtained by a rescaling *t* → *te*^βΔΕ^.

To compare the temperature-collapsed curves to our prediction of Eq. 6, we fit the parameters *p*_off_, τ_0_, $\bar{N}$ , and *N*_sat_ across multiple temperatures. The resulting fits, shown in Fig. 4, display a good agreement between the theory and experiments across up to four orders of magnitude in timescales. The fitted values of *N*_sat_ consistently increase with the estimated valence of the cross-linkers (Table 1), a trend that confirms our interpretation of the physical origin of *N*_sat_. The specific numerical values of these two quantities do not however match exactly, pointing to a possible gap in our understanding of the structure of each category of gels, and, in particular, the number of nearest neighbors of an individual cross-link, which remains debated for these types of gels (*34*–*36*). The possible clustering of cross-linkers may also influence this relation, as nanocage systems similar to ours (*37*) may form higher-order nanocage structures. While such complexities as well as possible imperfections in our fitting procedure complicate the literal interpretation of the fitted values of *N*_sat_, the global trend confirms our interpretation of its physical origin. The fit also supports the notion that the mean number of strands per superbond $\bar{N}$ accounts for the distribution of relaxation timescales in our gels. The Fe^3+^ gel thus displays an exponential relaxation consistent with $\bar{N}=N_{\text{sat}}=1$ . The higher-valence Pd_2_L_4_ and Pd_12_L_24_ systems have a complex relaxation at early times followed by an exponential behavior, as expected for $\bar{N}≃N_{\text{sat}}>1$ . As expected from our model, the crossover time τ_*N*_sat__ separating the two regimes is larger in the higher-valence Pd_12_L_24_ gel. Finally, the high-valence NP system shows an extended complex relaxation associated with $\bar{N}<N_{\text{sat}}$ , thus confirming that all the qualitative relaxation regimes discussed in the previous sections are experimentally relevant.

### Distribution of relaxation timescales

**Fig. 5.**
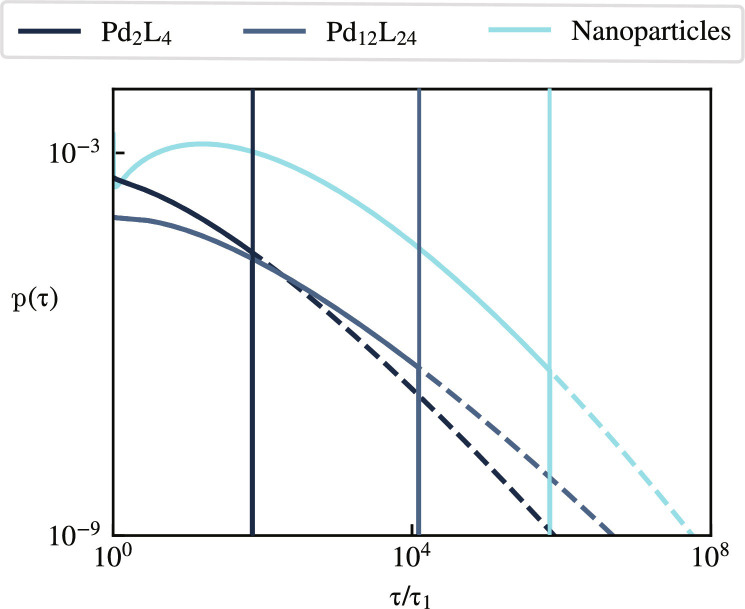
Distribution of relaxation times corresponding to the theoretical plots of Fig. 4. The distribution associated with the Fe^3+^ gel is a delta function for τ/τ_1_ = 1 and is thus not represented on this graph. The time distributions are given by a Poisson distribution cut for τ = τ_*N*_sat__ (vertical lines), as described in Eq. 6. The dotted lines represent what these distributions would be in the absence of this saturation.

To further visualize the differences between the responses of our gels, we plot the distributions of relaxation times *p*(τ) for our fitted model in Fig. 5. The Fe^3+^ gels, which relax according to a single exponential and whose *p*(τ) are therefore delta functions, are not represented there. In the Pd_2_L_4_ and Pd_12_L_24_ systems, a distribution characterized by an initially decreasing distribution of timescales is interrupted by a valence-dependent maximum relaxation time τ_*N*_sat__. That time is comprised within the range of timescales observed in Fig. 4, accounting for the crossover to an exponential relaxation within this range. In NP systems, by contrast, the crossover occurs much later and thus cannot be directly observed in experiments. In all cases, the precise form of the distribution of timescales used in the domain τ < τ_*N*_sat__ does not critically affect the predicted relaxation curves. We show in section S6 that replacing the Poisson distribution of Eq. 5 with other distributions with the same mean and variance lead to essentially indistinguishable predictions over experimentally observable timescales. This emphasizes the robustness of our predictions to the details of that choice of distribution. They are instead primarily determined by the mean and maximum superbond sizes, $\bar{N}$ and *N*_sat_.

In the limit of large $\bar{N}$ and even larger *N*_sat_, the complex relaxation phase of our model characterized by *t* < τ_*N*_sat__ may display analytical behaviors identical to some widely used rheological fitting functions. In this regime, the Poisson distribution *p*(*N*) of Eq. 5 goes to a Gaussian. Since, according to Eq. 4, the variable *N* is essentially the logarithm of the relaxation time τ for $\bar{N}\gg 1$ , this results in a log-normal distribution of relaxation timescales

p(τ)=12πN¯∣lnpoff∣τ×exp{−[lnτ+lnN¯τ1+lnpoff(N¯−1)]22N¯(lnpoff)2} (7)

This result adds additional insights to this widely used fitting functional form, as it allows us to relate the mean and variance of the distribution to the underlying cross-linker–scale parameters (*13*, *14*). It moreover offers a potential molecular-level justification for its use in describing the complex relaxation of systems with multivalent cross-links. In the alternative case where *p*(*N*) is a decaying exponential, our model results in power-law distributed relaxation timescales, and the stress response function takes the form

$$\sigma \left(t\right)\propto t^{-\gamma },\;\text{with}\;\gamma =\frac{1}{\overset{¯}{N}|\text{ln}p_{\text{off}}|}$$ (8)

This result may also be presented in terms of the dependence of the storage and loss moduli on the frequency ω in an oscillatory rheology experiment. We thus predict that for γ < 1

G′(ω)≈{ω2for ω≪τNsat−1ωγfor τNsat−1≪ω≪τ1−1ω0for τ1−1≪ω (9a)

G″(ω)≈{ω1for ω≪τNsat−1ωγfor τNsat−1≪ω≪τ1−1ω−1for τ1−1≪ω (9b)

The results for larger values of γ and detailed derivations of Eqs. 7 to 9 are shown in the Supplementary Materials. Again, this result has the potential to account for the power-law relaxation observed in many rheological systems (*10*–*12*), in addition to providing a link to their microscopic constituents. Overall, these results suggest a possible control of the system’s rheology through the characteristics of *p*(*N*), which could in turn be modulated through the spatial distribution of the polymer strands and the dispersity of the cross-linkers.

## DISCUSSION

Our simple model recapitulates a wide range of rheological behaviors in multivalent systems based on two key superbond parameters: the mean size $\bar{N}$ and the maximum size *N*_sat_. These respectively control the amplitude of the fluctuations in superbond size and the longest superbond relaxation timescale. Before the longest relaxation time, the system displays an increasingly nonexponential response for increasing $\bar{N}$ (Fig. 3B). Beyond it, it crosses over into exponential relaxation. In contrast with widely used phenomenological fitting parameters, our two variables yield reliable insights into the underlying microscopic dynamics, as demonstrated by the agreement of their fitting values with our a priori knowledge of four experimental systems covering a wide range of values of $\bar{N}$ and *N*_sat_.

Our model bears a mathematical similarity with standard random energy trap models (*38*). There, a long-tailed relaxation emerges from a short-tailed distribution of trap depths due to the exponential dependence of the relaxation times on the trap depths. Similarly, here, a nonexponential relaxation emerges from a short-tailed distribution of superbond sizes *N* (Eq. 5) thanks to the exponential dependence of τ*_N_* on *N* (Eq. 4). In contrast with trap models, however, our model does not predict a glass transition upon a lowering of temperature. It instead displays a simple Arrhenius time-temperature relation, consistent with the experimental collapses in the insets of Fig. 4. Another related relaxation process is manifested in multivalent polymer chains, which interact through multiple sticky domains. Recent data involving relatively short such chains suggest a relaxation mode governed by the simultaneous detachment of all stickers carried by a polymer, implying an exponential dependence of the relaxation time on valence similar to ours (*39*). The resulting macroscopic relaxation dynamics is however essentially exponential, either because of the relatively small valences involved or because valence fluctuations affect flexible polymers differently than our rigid cross-linkers. Such systems also display more complicated relaxation modes, from sticky Rouse-like diffusion to localized collective motions due to nanodomain clustering (*40*). The reasonable agreement between our estimated valences and the fitted values of *N*_sat_ suggests that while related, the relaxation mechanisms in our rigid cross-linker systems may be easier to relate to simple geometrical characteristics of their components.

Our model’s focus on the collective aspects of superbond breaking and the characteristics of the cross-linkers implies that it encloses most of the physics of the polymer strands within a few mesoscopic parameters, mainly τ_0_ and Δ*S*. Within our approach, the morphology of the polymer thus does not affect the form of our relaxation, although it may lead to a rescaling of the relaxation times of Eq. 4. This formulation remains valid as long as the length and concentration of the polymer strands is low enough that the strands do not become significantly entangled, which could spoil the Poissonian attachment/detachment process of Eq. 2. Even in this case, however, this equation may not be strictly valid, as the polymer layer in a superbond with many bound cross-linkers tends to be more compressed than in one with few. This effect should lead to a smooth (likely power law) dependence of ω^±^ on *N*, which would preserve the dominance of the much more abrupt exponential dependence of τ*_N_* on *N*. As a result, while such polymer brush effects could induce corrections in our estimations of the model parameters, the basic mechanism outlined here should still hold in their presence. The overall cross-linker and strand concentrations mainly influence two other model parameters, namely *N*_sat_, which depends on the number of neighbors of each cross-linker, and $\bar{N}$ , which counts the average number of available strands per superbond. Another assumption of our model is that neighboring superbonds do not exchange polymer strands. This is correct in our limit of short, strongly bound strands, which are unlikely to reach out to the next superbond or migrate toward it. While our experimental systems are not a priori guaranteed to be far into this asymptotic regime, the good agreement with our predictions suggests that it constitutes a reasonable approximation. Overall, it is worth keeping in mind that while many aspects of our model are idealized, its key result, namely the exponential dependence of τ*_N_* on *N*, is very robust to the introduction of more realistic, system-dependent features in the model. Such features thus leave our central conclusions about the influence of valence on the hierarchy of relaxation timescales intact, although they might modify our interpretation of certain model parameters. For instance, a possible clustering interaction between ligands on the surface of the cross-linker would imply that the energy Δ*E* is actually a sum of a ligand–cross-linker and a ligand-ligand interaction.

Our model reproduces several qualitative characteristics of the rheology of multivalent gels, such as the strong influence of the cross-linker valence, Arrhenius temperature dependence, and the transition between a nonexponential and an exponential regime at long times. Because of its simple, widely applicable microscopic assumptions, we believe that it could help shed light on and assist the design of a wide range of multivalent systems. Beyond composite gels, it could thus apply to RNA-protein biocondensates where multivalent interactions between proteins are mediated by RNA strands (*41*), as well as cytoskeletal systems where filaments linked to many other filaments display a slow relaxation reminiscent of that of our multivalent cross-linkers (*42*).

## METHODS

### Estimate of the amount of entanglements in our NP system

Our 10-kDa stars comprise 28 Kuhn segments per arm, each with length 0.76 nm (*43*), implying a radius *R* ≃ 4 nm. As the overall polymer density in our system is *c* = 10^−4^ mol ml^−1^, this implies that the concentration of our solution is lower than the overlap concentration

$$\frac{c}{c^{*}}=c\frac{4\pi R^{3}}{3}≃0.64$$ (10)

and therefore that our initial solution is not entangled.

Now considering the situation within a single superbond between two 7-nm NP as opposed to the average situation within the solution, the polymer size and the fitted values of Table 1 suggest that a superbond contains the equivalent of $\bar{N}=14$ bifunctional polymer strands in a volume of the order of that of a particle. This yields a packing length (typical distance between entanglements) *p* ≃ 2 nm (*44*). This suggests that each chain is entangled once or a few times with its neighbors, which could slow down the system’s relaxation dynamics, but which we do not expect to impose topological constraints strong enough that the polymer’s reptation time would differ from its Rouse time by orders of magnitude.

### Materials

4-arm PEG bis(acetic acid *N*-succinimidyl) ester (4-arm PEG-NHS) (*M*_W_ = 10 kDa) and 1-arm PEG-NHS (*M*_W_ = 2000 Da) were purchased from JenKem Technology. Sodium sulfate (Na_2_So_4_), sodium nitrite (NaNO_2_), iron(III) acetylacetonate [(Fe(acac)_3_], hydrochloric acid (HCl), dopamine hydrochloride, triethylamine (TEA), *N*-methylmorpholine (NMM), dimethyl sulfoxide (DMSO), methanol (MeOH), ethanol (EtOH), dichloromethane (DCM), *N*,*N′*-dimethylformamide (DMF), diethyl ether (Et_2_O), and chloroform (CHCl_3_), were purchased from Sigma-Aldrich. All chemicals were used without further purification.

### Synthesis of 1-arm PEG-catechol 

Two hundred and twenty eight milligrams of dopamine hydrochloride is neutralized for 15 min with 0.3 ml NMM in 7.5 ml of dry DMF under N_2_ atmosphere. Then, 1 g of mPEG-NHS (*M*_W_ = 2000 Da) dissolved in 7.5 ml of DMF is added, and the mixture is stirred with N_2_ protection at room temperature for 24 hours. The reacted solution is acidified by adding 15 ml of 1 M HCl (aq), and the product is extracted with CHCl_3_ three times. The organic layers are pooled together and dried with NaSO_4_, and solvent is removed by rotary evaporation. Last, the product concentrate is precipitated in cold Et_2_O (−20°C), filtered, and dried. 1H nuclear magnetic resonance (NMR) (300 MHz, D_2_O) δ parts per million (ppm): 6.7 to 6.8 (m, 3H, aromatic), 3.3 to 4.0 (m, −O − CH_2_ − CH_2_−), 3.4 (t, 2H, CH_2_ adjacent to aromatic ring), 2.7 (t, 2H, ─CH_2_─NH─CO─).

### Synthesis of 4-arm PEG-nitrocatechol

One hundred and seventy-eight milligrams of nitrodopamine hydrogen sulfate is neutralized for 15 min with 110 μl of NMM in 4 ml of dry DMF under N_2_ atmosphere. Then, 1 g of 4-arm PEG-NHS (*M*_W_ = 10 kDa) dissolved in 4 ml of DMF is added, and the mixture is stirred with N_2_ protection at room temperature for 24 hours. The reacted mixture is mixed with 15 ml of 1 M HCl_(*aq*)_, dialyzed with water (MWCO = 3500 Da) for 2 days (water exchanged for more than five times), and freeze-dried. 1H NMR (300 MHz, D_2_O) δ (ppm): 7.6 (m, 1H, aromatic), 6.7 (m, 1H, aromatic), 3.6 to 3.9 (m, ─O─CH_2_─CH_2_─), 3.5 (t, 2H, CH_2_ adjacent to aromatic ring), 3.1 (t, 2H, ─CH_2_─NH─CO─).

### Synthesis of Fe_3_O_4_ NPs

Bare Fe_3_O_4_ NPs are synthesized following previously reported methods (*44*). One hundred milligrams as-synthesized NPs are redispersed in 80 ml of 1:1 (v/v) solution of CHCl_3_ and DMF, and 100 mg 1-arm PEG-C is added. The mixture is homogenized and equilibrated by pulsed sonication (pulse: 10 s on +4 s off; power: 125 W) for 1 hour. Then, the mixture is centrifuged at 10,000 rpm for 10 min to remove any aggregates and rotary evaporated at 50°C, 30 mbar to remove CHCl_3_. Then, the NP solution is precipitated in 150 ml cold Et_2_O (−20°C). The precipitate is redispersed in H_2_O and freeze-dried. The resulting NPs are 7 nm in diameter.

### Preparation of the Fe3^+^-NC gels

Preparation procedure is similar to a previously reported protocol (*45*), except that the gel is made in DMSO instead of H_2_O. Fifty microliters of 4-arm PEG-NC solution (200 mg/ml) in DMSO is mixed with 16.7 μl of 80 mM FeCl_3_ solution in DMSO (ligand: Fe^3+^ molar ratio of 3:1). Then, 33.3 μl of DMSO and 13.8 μl of TEA is added to facilitate deprotonation, and a gel is formed.

### Preparation of the Pd_2_L_4_ gels

The synthesis of polymer and gel preparation procedures for P_2_L_4_ is the same as a reported protocol (*33*) with minor modifications. The annealing of the Pd_2_L_4_ polyMOC gel was done at 60 Â°C for 1 hours instead of 80°C for 4 hours and 1.05 equivalent of Pd(NO_3_)_2_. 2 H_2_O (relative to bifunctional polymer ligand) was used instead of 1 equivalent.

### Preparation of the Pd_12_L_24_ gels

The synthesis of polymer and gel preparation procedures for polyMOC is the same as a reported protocol (*33*).

### Preparation of the NP gels

Preparation procedure is the same as the reported protocol (*46*). Briefly, PEGylated Fe_3_O_4_ NPs (equivalent to 20-mg Fe_3_O_4_ core) and 20-mg 4-arm PEG-NC are mixed in a 0.2 M HCl aqueous solution. The solution mixture (pH 2) is transferred into a mold and sealed, and a solid gel is obtained after curing in a 50°C oven for 24 hours.

### Rheology

Stress relaxation measurements are done on an Anton Paar rheometer with parallel plate geometry (10-mm diameter flat probe for NP gels and polyMOC gels and 25-mm diameter cone probe for Fe^3+^ gels). All tests are done immediately after transferring the gel sample onto the sample stage. A Peltier hood is used for all experiments to control the measurement temperature and prevent solvent evaporation. H_2_O-based samples are furthermore sealed with mineral oil before experimentation to reduce the evaporation rate. Relaxation tests were performed by applying a γ = 0.005 step strain for the NP gel and γ = 0.02 step strain for the other three systems.

## Supplementary Materials

### This PDF file includes:

Figs. S1 to S8 Table S1 Sections S1 to S9
