# Supplementary material for: Valence can control the nonexponential viscoelastic relaxation of multivalent reversible gels
Source: Sci Adv. 2024 May 15;10(20):eadl5056. doi: 10.1126/sciadv.adl5056 (PMC11095449; doi:10.1126/sciadv.adl5056)
Supplement: Supplementary file 1 — Figs. S1 to S8 Table S1 Sections S1 to S9 [file sciadv.adl5056_sm.pdf]

Science Advances
AAAS

Supplementary Materials for

Valence can control the nonexponential viscoelastic relaxation of multivalent reversible gels

Hugo Le Roy *et al.*

Corresponding author: Hugo Le Roy, h.leroy@epfl.ch; Martin Lenz, martin.lenz@universite-paris-saclay.fr

Sci. Adv. **10**, eadl5056 (2024)
DOI: 10.1126/sciadv.adl5056

This PDF file includes:

Figs S1 to S8
Table S1
Sections S1 to S9

S1. DISTRIBUTION OF SUPERBOND BREAKING TIME AND DERIVATION OF τ_N

Here we show that the survival probability for the detachment of a superbond (illustrated in main text Fig. 2) containing many polymer strands ($N \to \infty$) asymptotically goes to $S(t) = e^{-t/\tau_N}$, where τ_N is given by Eq. (4) of the main text. We first consider a general one-step process and derive the basic recursion equation used throughout the proof in Sec. S1.1. We solve the recursion in Sec. S1.2 and express the generating function of $S(t)$ as a double sum. In Sec. S1.3, we apply the resulting formula to our particular problem and take the continuum limit of the second sum. Finally, we compute both sums in the $N \to \infty$ limit in Sec. S1.4. Our derivation is adapted from the calculation presented in the appendix of Ref.[32]

S1.1. Backward Kolmogorov equation for the generating function of $S(t)$

We consider a one-step process, *i.e.*, a stochastic process consisting of transitions between consecutive discrete states on a line, with transition rates r_n and g_n illustrated in Fig. S1(a). We denote the probability for the particle to be in state k at time t after starting in state n at time 0 by $P(k, t|n)$. We assume an absorbing boundary condition in 0 and a reflecting boundary condition in N, *i.e.*,

$$\forall n \in [1..N] \quad P(0, t|n) = 0, \qquad r_N = 0. \tag{S1}$$

The backward Kolmogorov equation for our process reads [31]

$$\frac{\mathrm{d}P}{\mathrm{d}t}(k, t|n) = g_n[P(k, t|n + 1) - P(k, t|n)] - r_n[P(k, t|n) - P(k, t|n - 1)]. \tag{S2}$$

We define the survival probability and its generating function (Laplace transform), respectively as

$$S_n(t) = \sum_{k=1}^{N} P(k, t|n), \quad h_n(\alpha) = \int_0^{+\infty} S_n(t)e^{-\alpha t}\,\mathrm{d}t. \tag{S3}$$

Inserting these definitions into Eq. (S2) yields

$$\alpha h_n(\alpha) - 1 = g_n[h_{n+1}(\alpha) - h_n(\alpha)] - r_n[h_n(\alpha) - h_{n-1}(\alpha)], \tag{S4}$$

which we endeavor to solve for $h_n(\alpha)$ in the following.

S1.2. Sum equation for the generating function

We define a rescaled current between sites $n - 1$ and n

$$\Delta_n = \begin{cases} r_n \left(\prod_{i=n}^{N-1} \frac{r_{i+1}}{g_i}\right) [h_n - h_{n-1}] & \text{for } n < N \\ r_N[h_N - h_{N-1}] & \text{for } n = N \end{cases}. \tag{S5}$$

This allows us to turn the two-step recursion of Eq. (S4) into one with only one step:

$$\Delta_n = \begin{cases} \Delta_{n+1} + \left(\prod_{i=n}^{N-1} \frac{r_{i+1}}{g_i}\right) [1 - \alpha h_n] & \text{for } n < N \\ 1 - \alpha h_N & \text{for } n = N \end{cases}, \tag{S6}$$

which can easily be summed as

$$\Delta_n = \left[\sum_{j=n}^{N-1} \left(\prod_{i=n}^{N-1} \frac{r_{i+1}}{g_i}\right) (1 - \alpha h_j)\right] + 1 - \alpha h_N. \tag{S7}$$

We now invert Eq. (S5) and use Eq. (S7) to express the finite difference $(h_n - h_{n-1})$. We further use the property that $h_m = h_0 + \sum_{n=1}^{m}(h_n - h_{n-1})$ and recognize that $h_0 = 0$ due to Eq. (S1) to obtain

$$h_m = \sum_{n=1}^{m} \frac{1}{r_N} \left(\prod_{i=n}^{N-1} \frac{g_i}{r_{i+1}}\right) \left\{ \left[\sum_{j=n}^{N-1} \left(\prod_{i=n}^{N-1} \frac{r_{i+1}}{g_i}\right) (1 - \alpha h_j)\right] + 1 - \alpha h_N \right\}. \tag{S8}$$

(a)

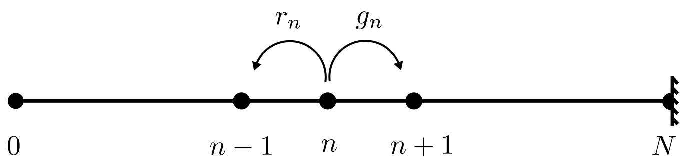

(b)

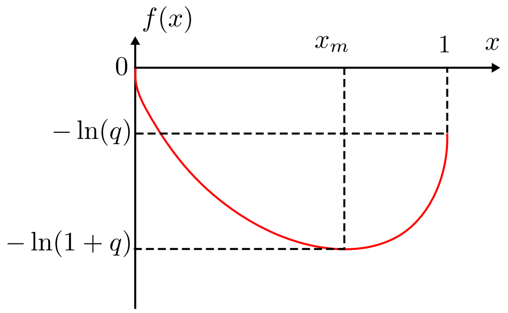

FIG. S1. **Superbond detachment as a Kramers-like barrier-crossing problem.** (a) Definition of the rates of the one-step process. (b) Profile of the pseudo-free energy defined in Eq. (S13). Superbond detachment requires the system to fluctuate out of the free energy well to the $x = 0$ absorbing state, with $1/N$ playing the role of a temperature.

S1.3. Application and continuum limit

Using the mean detachment time of a polymer strand (denoted as ω_- in the main text) as our unit of time and defining $q = \omega_+/\omega_-$, the model of the main text implies

$$\forall n \in [1..N] \quad r_n = n, \quad g_n = (N-n)q, \tag{S9}$$

which we insert into Eq. (S8) to obtain

$$h_n = \sum_{j=1}^{n} \frac{1}{j\binom{N}{j}q^j} \sum_{i=j}^{N} \binom{N}{i} q^i (1 - \alpha h_i). \tag{S10}$$

In Eq. (S10), the outermost sum is dominated by the very small values of j in the limit $N \to \infty$. We thus need only consider small values of j when computing the innermost sum, which happens to be dominated by a value of i far from the edges of the $[1..N]$ interval. We can thus take its continuum limit. Using Stirling's formula, we obtain

$$h_n \underset{N\to\infty}{\sim} \sum_{j=1}^{n} \frac{1}{j\binom{N}{j}q^j} \int_0^1 \sqrt{\frac{N}{2\pi x(1-x)}} e^{-Nf(x)} [1 - \alpha h(x,\alpha)] \, \mathrm{d}x, \tag{S11}$$

where we have defined the continuum version of our generating function though $h(x, \alpha) = h_{Nx}(\alpha)$, as well as the pseudo free energy of the system

$$f(x) = x \ln x + (1-x)\ln(1-x) - x \ln q. \tag{S12}$$

This free energy has a single minimum in $x_m = q/(1 + q)$ with a locally parabolic structure given by

$$f(x) = -\ln(1+q) + \frac{(1+q)^2}{2q}(x - x_m)^2 + \mathcal{O}(x - x_m)^3, \tag{S13}$$

which we illustrate in Fig. S1(b). The problem at hand is exactly analogous to a Kramers escape problem from the bottom of this minimum to the $n = 0$ boundary condition, with $N \to \infty$ playing the role of the low-temperature limit.

S1.4. Asymptotic simplifications

Using the Kramers analogy to our advantage, we compute the integral of Eq. (S11) using a saddle-point approximation. We thus find that for any $x \in]0,1[$:

$$h(x,\alpha) \underset{N\to\infty}{\sim} (1+q)^N [1-\alpha h(x_m,\alpha)] \sum_{j=1}^{Nx} \frac{q^{-j}}{j\binom{N}{j}}. \tag{S14}$$

Using Stirling's formula for small values of j reveals that the argument of the sum in Eq. (S14) goes as $(j-1)! \times (Nq)^{-j}$. Therefore, the terms of the sum are simply the terms in an expansion in powers of N. We keep only the lowest-order term to find

$$\forall x \in]0,1[\quad h(x,\alpha) \underset{N\to\infty}{\sim} \tau_N [1-\alpha h(x_m,\alpha)]. \tag{S15}$$

where

$$\tau_N = \frac{(1+q)^N}{Nq} \tag{S16}$$

is the dimensionless version of the mean first-passage time presented in Eq. (4) of the main text.

Setting $x = x_m$, Eq. (S15) implies

$$h(x_m,\alpha) \underset{N\to\infty}{\sim} \frac{1}{\alpha + \tau_N^{-1}} \quad \Leftrightarrow \quad S_{Nx_m}(t) \underset{N\to\infty}{\sim} e^{-t/\tau_N}. \tag{S17}$$

Finally, using Eq. (S15) again yields

$$\forall x \in]0,1[\quad S_{Nx}(t) \underset{N\to\infty}{\sim} -\tau_N \frac{\mathrm{d}S_{Nx_m}}{\mathrm{d}t}(t) = e^{-t/\tau_N}, \tag{S18}$$

which is the exponential distribution presented in the main text.

S2. LINK BETWEEN α AND $N_{\mathrm{sat}}/\bar{N}$

Here establish the connection between the stretch exponent α and the values of $N_{\mathrm{sat}}/\bar{N}$ shown in Fig. 4 of the main text. To mimic the observation of an experimental step strain over a finite time window, we focus our attention on the time interval between $t = 0$ and $t = \tau_{90}$, where τ_{90} is the time required to relax 90% of the stress, *i.e.*, $\sigma(\tau_{90}) = 0.1 \times \sigma(0)$. We plot the relaxation curve given by Eq. (6) of the main text over this time window, then perform a least-squares fit using a stretched exponential [Eq. (1) of the main text] with α and τ as fitting parameters. As shown in Fig. S2, the agreement is excellent for a large majority of the parameters used. The corresponding value of the fitting parameters (τ and α) for a broader variety of $\bar{N}$ and N_{sat} is also provided in Fig. S3. This suggests that experimental curves that are well fitted by a stretched exponential could be equally well described by our model.

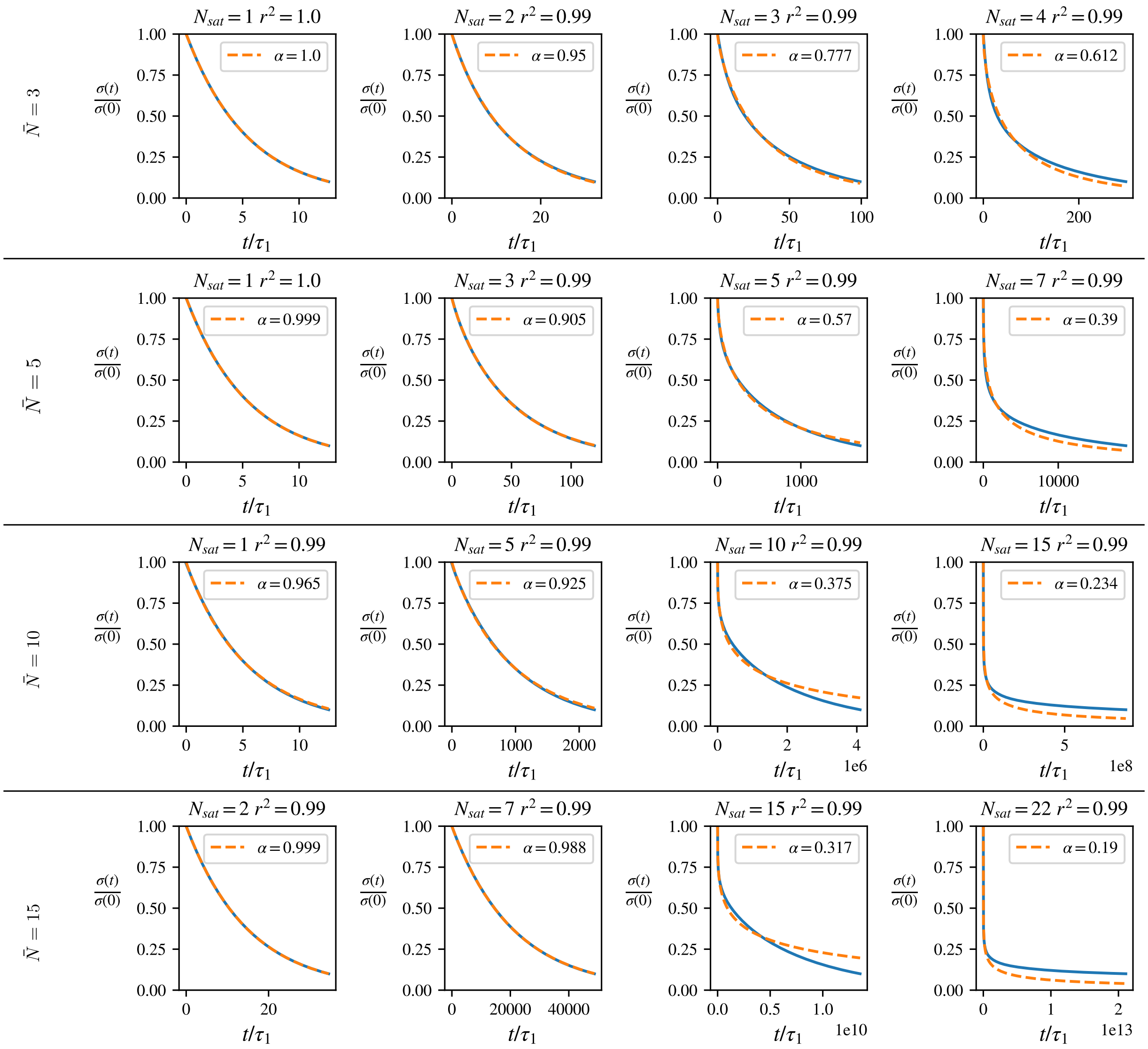

FIG. S2. **Illustration of the similarity of our modeled stress response function with a stretched exponential.** We plot the relaxation modulus computed using Eq. (6) of the main text for $\bar{N} \in [3, 5, 10, 15]$. For each value of $\bar{N}$, we plot four values of N_{sat}, namely $N_{\mathrm{sat}} = 0.1\bar{N}$, $0.5\bar{N}$, $\bar{N}$ and $1.5\bar{N}$, $p_{\mathrm{off}} = 0.2$. Each plot also mentions the value of the fitted stretch exponent α and the correlation coefficient r^2.

S3. TIME-TEMPERATURE COLLAPSE

Here we describe the procedure used to determine the binding energy ΔE in the experimental systems discussed in the main text. Equation (4) of the main text implies that the temperature dependence of the stress response function can be eliminated by expressing it as a function of the rescaled time $\tilde{t} = te^{\beta\Delta E}$. This should cause the relaxation curves of a given system at different temperatures to collapse.

For each type of ligand, we have 5 datasets showing the stress relaxation function as a function of time at each different temperature $\{T^{(\alpha)}\}_{\alpha\in[0,4]} = \{25°\mathrm{C}, 35°\mathrm{C}, 45°\mathrm{C}, 55°\mathrm{C}, 65°\mathrm{C}\}$. To enable the comparison between time-rescaled datasets, we first define an interpolating function for the stress relaxation function at each temperature used. We thus compute the set of interpolating coefficients $\left\{p_k^{(\alpha)}\right\}_k$ by perform a least-square fit of the following rational function

$$P^{(\alpha)}(t) = \sum_{k=-3}^{10} p_k^{(\alpha)} t^k, \tag{S19}$$

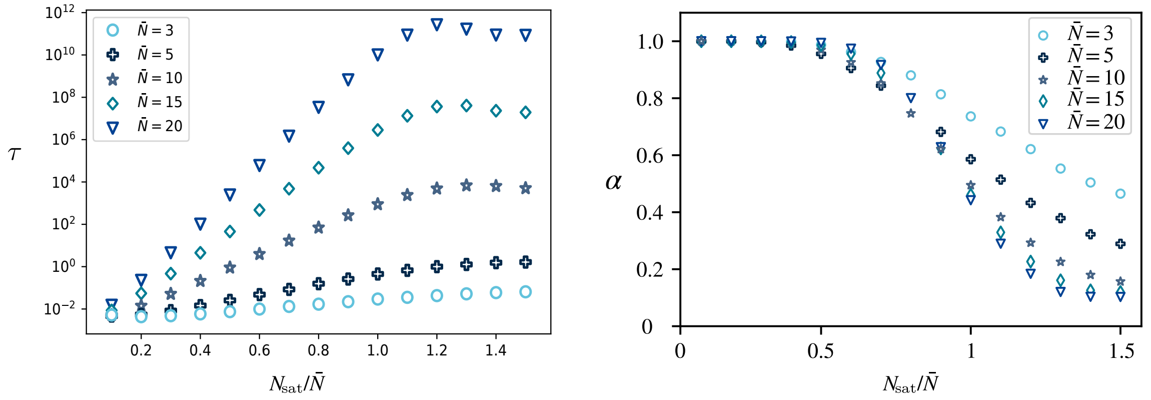

FIG. S3. **Best fit values of the stretched exponential**. We fit the parameters τ and α for a range of values of $\bar{N}$ and $N_{\rm sat}$. The right-hand-side panel is identical to Fig. 4 of the main text.

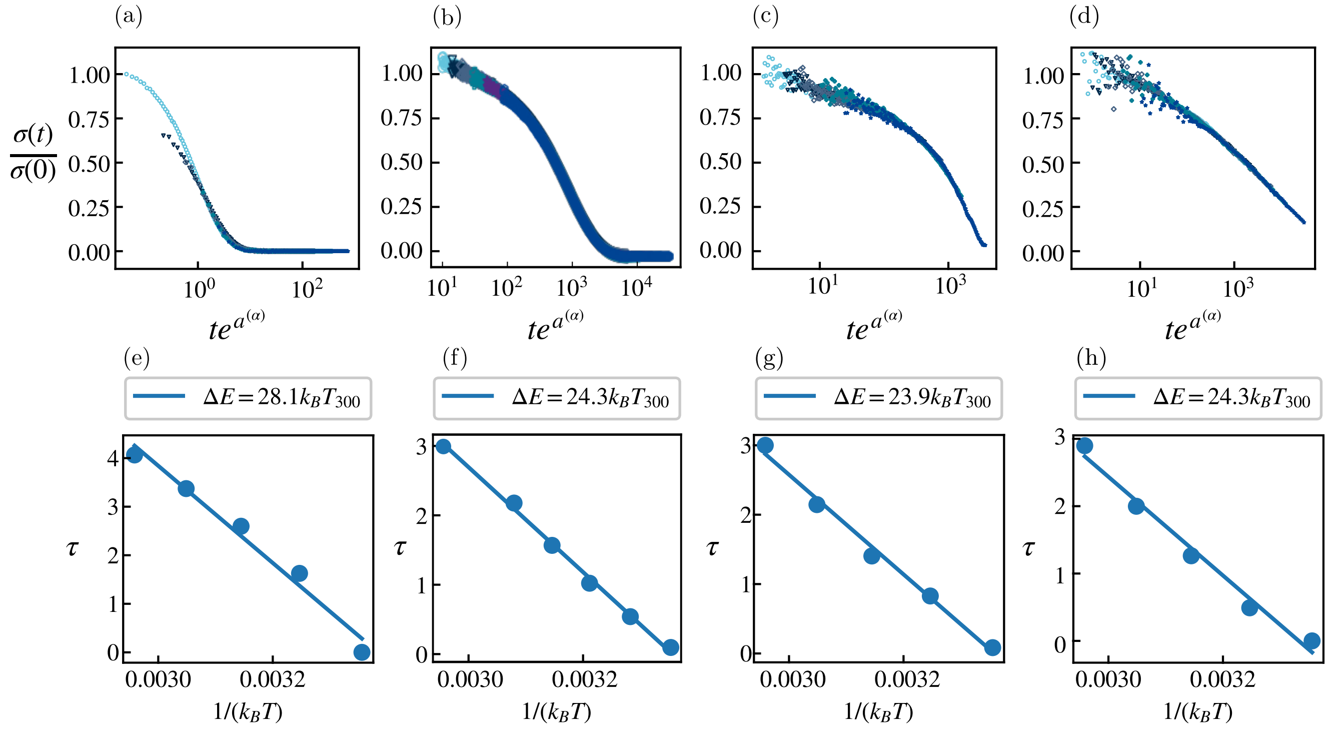

FIG. S4. **Collapse of the relaxation modulus.** (a-c) Collapsed relaxation modulus of Fe^{3+} ions, $Pd_{12}L_{24}$ nanocages and nanoparticles respectively after the rescaling of the time for an optimized collapse . The curves are represented on a log-lin coordinate system, but the collapsing procedure is performed on a lin-lin scale . (d-f) corresponding rescaling parameters as a function of $1/(k_BT)$ the inverse temperature. The slope of the line is $-\Delta E$ and the legend gives the value of ΔE in k_BT unit at $300K$.

to the datapoints $\left\{t_i^{(\alpha)}, \frac{\sigma^{(\alpha)}(t_i)}{\sigma^{(\alpha)}(0)}\right\}_i$. We furthermore define the interval of definition of $P^{(\alpha)}(x)$ as the range over which data is available, *i.e.*, $I_{P^{(\alpha)}} = \left[0, \max_i t_i^{(\alpha)}\right]$.

We then perform the collapse of the $\{T^{(\alpha)}\}_{\alpha\in[1,4]}$ interpolated curves onto the $T^{(0)}$ curve. To this effect we define the set of rescaling coefficients $\{a^{(\alpha)}\}_{\alpha\in[1,4]}$ and performs a separate time rescaling for each temperature: $\tilde{t} = te^{a^{(\alpha)}}$. For each $\alpha \in [1,4]$, we optimise the semidistance

$$D(P,Q) = \int_{I_Q \cap I_P} [P(t) - Q(t)]^2 \mathrm{d}t, \tag{S20}$$

between the functions $t \to P^{(0)}(t)$ and $t \to P^{(\alpha)}(te^{a^{(\alpha)}})$ with respect to $a^{(\alpha)}$. The resulting collapsed curves are shown

in Fig. S4 (a,b,c). The optimal rescaling coefficients are plotted as a function of the inverse temperature $1/k_BT$ in Fig. S4 (d,e,f). Consistent with the time-temperature collapse hypothesis, this dependence is affine, and we use the slope of the best fitting line as our value of the binding energy ΔE.

S4. FIT OF THE STRESS RELAXATION FUNCTION TO OUR THEORETICAL PREDICTION

In the main text, we fit the experimental curves with the stress relaxation function predicted by our model. We then represent them on a log-lin scale to allow the simultaneous visualization of short and long time scales. To demonstrate the robustness of our fits, in Fig. S5 we replot these curves in a lin-lin-scale, as well as a lin-log scale that emphasizes intervals of exponential relaxation as straight lines.

S5. RATIONALIZATION OF THE POISSON DISTRIBUTION OF THE SUPERBOND SIZE $p(N)$

The polymers used in our experiments are 4-arms polyethylene glycol (PEG). At the end of each arm is a nitrocatechol ligand that allows crosslinker binding. In our model, we assume that the ends of a polymer are always attached to a ligand. For this reason, the diffusion of such a polymer over a distance comparable to the polymer size occurs on a time scale comparable to the time required to rearrange the bonds between crosslinkers, which corresponds to the time required for the relaxation of the stress in the system. Let us consider that the 4-arm PEG are able to diffuse over a volume v during the time of the experiment.We model the spreading of the polymers in the system by discretizing the system into small boxes of volume v between which no polymer exchange occurs over the duration of the experiment. As a result the distribution of the polymers over the boxes is due to the initial preparation of the system. We assume that this processes places each polymer in a random box with equal probability. As a result, the probability that a specific box contains n polymers is given by a Poisson distribution:

$$P(n) = e^{-\rho_{\mathrm{PEG}} v} \frac{(\rho_{\mathrm{PEG}} v)^n}{n!}, \tag{S21}$$

where ρ_{PEG} is the average concentration of PEG in the system, and $v\rho_{\mathrm{PEG}}$ is the mean (over the system) number of PEG in a box of volume v. Equation (S21) is the basis for Eq. (5) of the main text.

S6. EXPERIMENTAL FIT USING ALTERNATIVE DISTRIBUTIONS OF N

To demonstrate the robustness of our model, we perform the fit of the experimental data using probability distribution different from the Poisson distribution of Eq. (5) of the main text. For this, we first define three new distributions with the same mean (and when possible the same variance) than the one of Eq. (5) of the main text and keep the saturation value identical to the one in the main text. We first use a rectangular distribution :

$$\text{if } \bar{N} - \sqrt{3\bar{N}} < 0 \quad p_{\mathrm{rectangular}}(N) = \begin{cases} \frac{1}{\bar{N}+\sqrt{3\bar{N}}} & \text{for } N \in [0, \bar{N} + \sqrt{3\bar{N}}] \\ 0 & \text{otherwise} \end{cases} \tag{S22a}$$

$$\text{if } \bar{N} - \sqrt{3\bar{N}} > 0 \quad p_{\mathrm{rectangular}}(N) = \begin{cases} \frac{1}{2\sqrt{3\bar{N}}} & \text{for } N \in [\bar{N} - \sqrt{3\bar{N}}, \bar{N} + \sqrt{3\bar{N}}] \\ 0 & \text{otherwise} \end{cases}, \tag{S22b}$$

a triangular distribution:

$$p_{\mathrm{triangular}}(N) = \begin{cases} \frac{N}{\bar{N}^2} & \text{for } N \in [0, \bar{N}] \\ \frac{2}{\bar{N}} - \frac{N}{\bar{N}^2} & \text{for } N \in [\bar{N}, 2\bar{N}] \\ 0 & \text{otherwise} \end{cases}, \tag{S23}$$

and a linear distribution:

$$p_{\mathrm{linear}}(N) = \begin{cases} \frac{8N}{9\bar{N}^2} & \text{if } N \in [0, 3\bar{N}/2] \\ 0 & \text{otherwise} \end{cases}. \tag{S24}$$

In each of these cases, we introduce a truncation at the maximum superbond size in the same way as in Eqs. (5-6) of the main text (with discrete sums replaced by integrals). The resulting fits are displayed in Fig. S6, and the

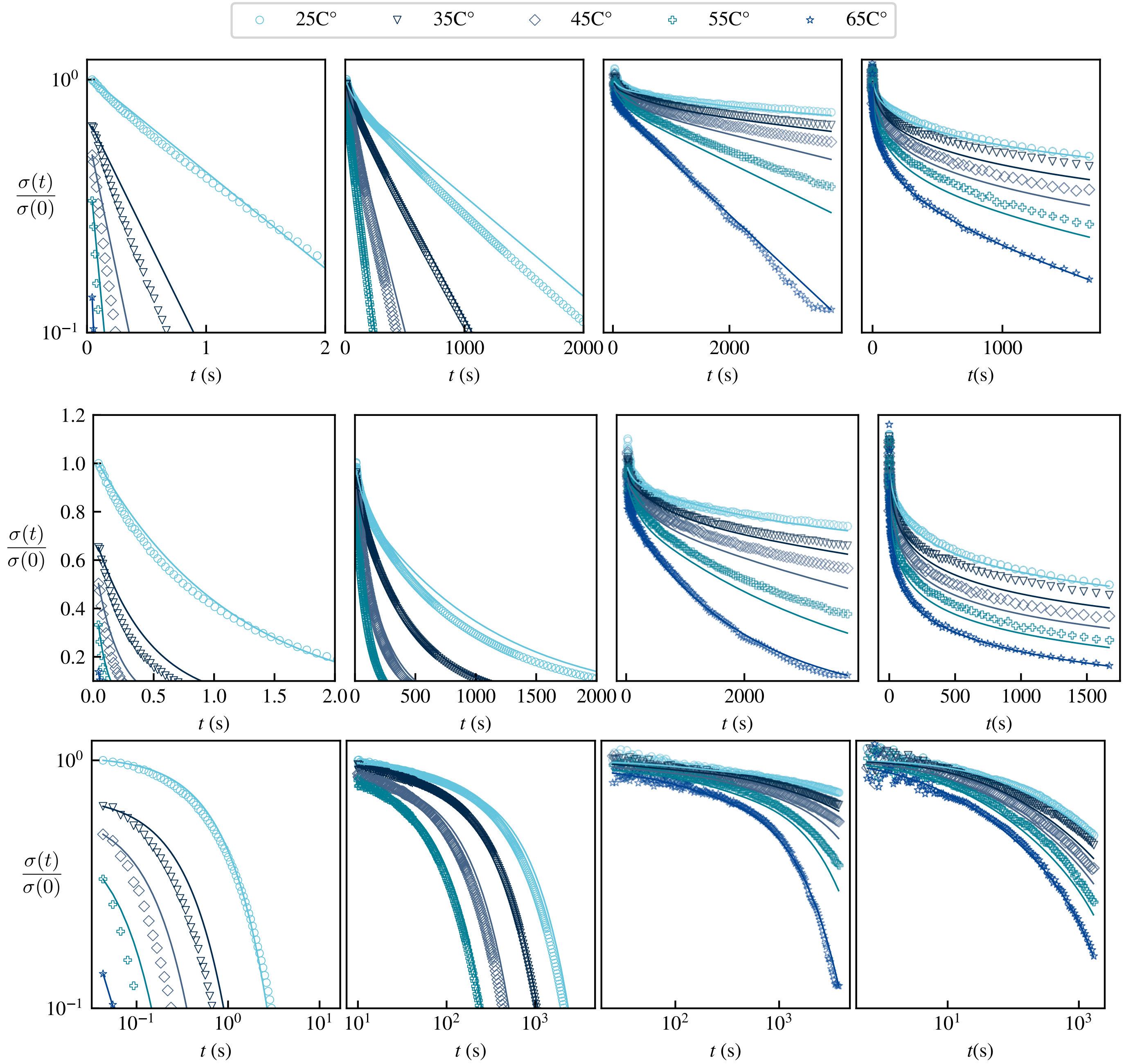

FIG. S5. **Fits of the experimental curves**. Respectively lin-lin and lin-log and log-log representation of Fig. 5 in the main text.

corresponding value of the fitting parameters are given in Table S1. The fitting curves and fitting parameters remain close to the ones obtained in the main text, implying that our specific choice of a Poisson distribution of superbond sizes is not critical for the validity of our results.

S7. DERIVATION OF THE LOG-NORMAL PROBABILITY DISTRIBUTION.

Here we derive Eq. (7) of the main text. Considering the Eq. (5) of the main text without the cutoff due to the valence, the distribution of single strands in a superbond becomes a simple Poisson distribution of mean $\bar{N}$. A random number drawn from such a distribution can also be considered as the sum of $\bar{N}$ independent variables drawn from a

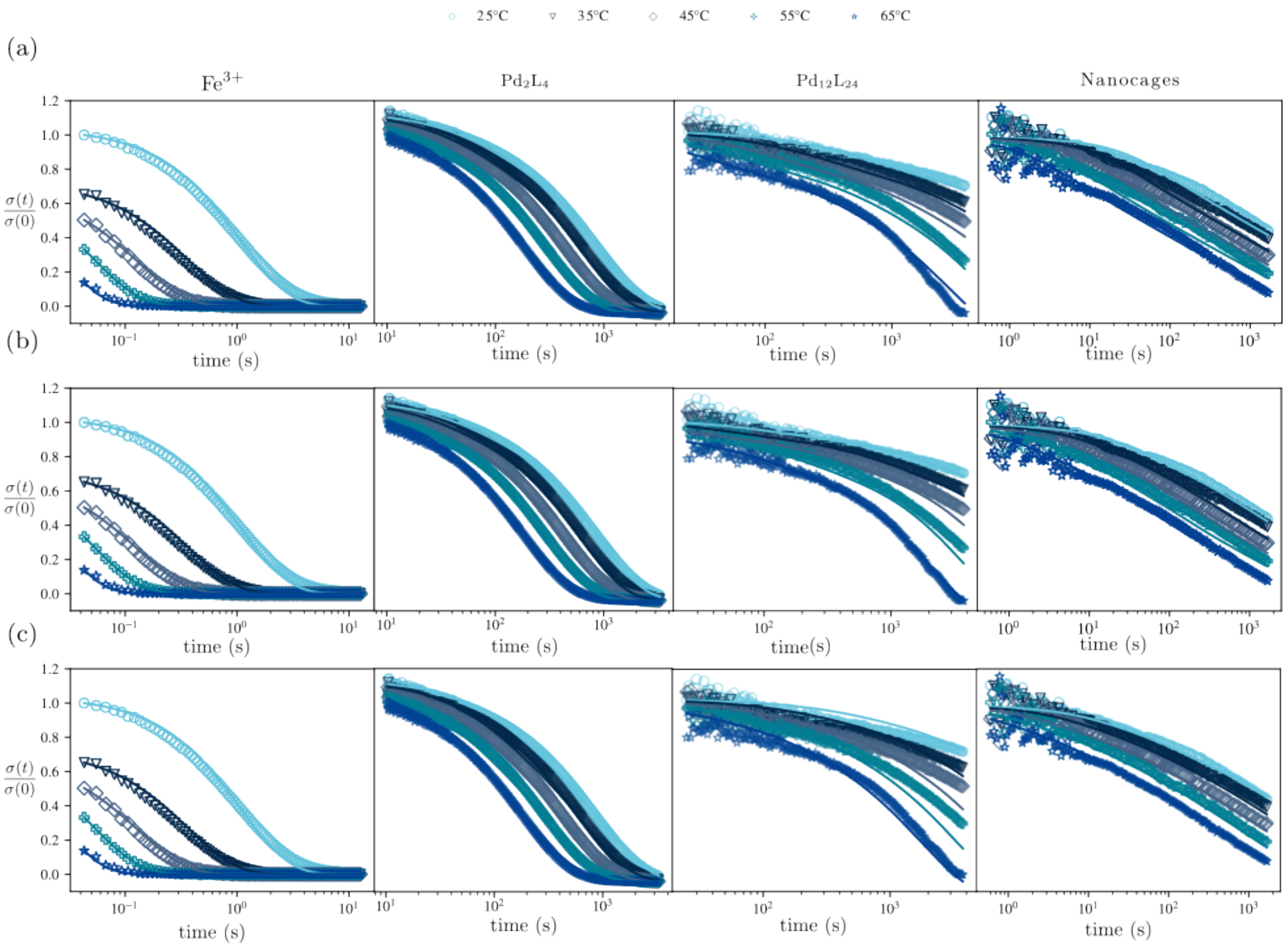

FIG. S6. **Fit of the experimental data using alternative probability distribution of N.** Respectively Fe^{3+}, nanoparticles and nanocage relaxation curves fitted using (a) a uniform distribution, (b) a triangular distribution and (c) using a linear distribution.

	parameter	Fe^{3+}	Pd_2L_4	$Pd_{12}L_{24}$	nanoparticles
uniform	$\mathbf{p_{off}}$	0.08	0.07	0.06	0.3
	τ_1(s)	0.86	122	41	11
	$\bar{\mathbf{N}}$	1	2	4	7
triangular	$\mathbf{p_{off}}$	0.09	0.02	0.03	0.5
	τ_1(s)	0.86	4	83	13
	$\bar{\mathbf{N}}$	1	3	4	5
linear	$\mathbf{p_{off}}$	0.08	0.04	0.07	0.24
	τ_1(s)	0.86	62	$3.9\ 10^2$	5.7
	$\bar{\mathbf{N}}$	1	2	3.3	6.3

TABLE S1. Value of the fitting parameters for the three alternative distributions of N indicated in the left column.

Poisson distribution of mean 1. According to the central limit theorem, the probability distribution of the sum of $\bar{N}$ independent random numbers converges to a normal distribution of mean $\bar{N}$ and variance $\sqrt{\bar{N}}$ for $\bar{N} \gg 1$. Moreover, as the main dependence of τ_N on N is exponential, in the large-$\bar{N}$ limit replacing the factor of N preceding $p_{\rm off}^N$ by the typical value $\bar{N}$ induces only a small (logarithmic) error. We thus approximate: $\tau_N/\tau_1 \sim \exp[N \log(p_{\rm off})]/\bar{N}$, and

write:

$$N = -\frac{\ln(\bar{N}\tau_N/(\tau_1 p_{\rm off}))}{\ln(p_{\rm off})}. \tag{S25}$$

Treating N as a continuous variable when computing the stress relaxation function allows us to change variable:

$$\begin{aligned}\frac{G(t)}{G(0)} &= \sum_N p(N)\exp(-t/\tau_N)\\ &\approx \int_N p(N)\exp(-t/\tau_N)dN \\ &= \int_\tau p(\tau)\exp(-t/\tau)d\tau.\end{aligned} \tag{S26}$$

Where we used in the last line the identity:

$$p(\tau)d\tau = p(N)dN, \tag{S27}$$

Substituting N to τ in the approximate normal distribution of N finally gives a log-normal distribution of relaxation time presented in Eq. (7) of the main text.

S8. DERIVATION OF THE POWER LAW RELAXATION

Here we derive Eq. (8) of the main text. As discussed in the main text, substituting the superbond size distribution Eq. (5) of the main text for an exponential distribution

$$p(N) = \left(1 - e^{-1/\bar{N}}\right) e^{-N/\bar{N}} \tag{S28}$$

yields a power-law relaxation regime provided that $\bar{N} \gg 1$, as shown in Fig. S7. Here we compute the value of the relaxation exponent.

Since Eq. (S28) does not saturate at a finite $N = N_{\rm sat}$, Eq. (6) of the main text becomes

$$\frac{\sigma(t)}{\sigma(0)} = \sum_{N=1}^{+\infty} \frac{p(N)}{1-p(0)} e^{-t/\tau_N} \qquad \text{with} \qquad \tau_N = \frac{\tau_0 e^{\beta\Delta E}}{N p_{\rm off}^N}. \tag{S29}$$

We employ the same approximation as in Sec. S7, which captures the dominant exponential relationship between τ and N:

$$\tau_N \simeq \frac{\tau_0 e^{\beta\Delta E}}{\bar{N} p_{\rm off}^N}. \tag{S30}$$

We also take the continuum limit of the sum of Eq. (S29) as is appropriate for large $\bar{N}$. Defining the dimensionless time $\tilde{t} = t\bar{N}/\tau_0 e^{\beta\Delta E}$, this yields

$$\frac{\sigma(t)}{\sigma(0)} \underset{\bar{N}\gg 1}{\sim} \int_0^{+\infty} p(N) e^{-\tilde{t} p_{\rm off}^N}\, \mathrm{d}N. \tag{S31}$$

We next change our integration variable to $\tilde{\tau} = p_{\rm off}^N$ to find

$$\frac{\sigma(t)}{\sigma(0)} \underset{\bar{N}\gg 1}{\sim} \int_1^{+\infty} \gamma \tilde{\tau}^{-(1+\gamma)} e^{-\tilde{t}/\tilde{\tau}}\, \mathrm{d}\tilde{\tau} \underset{\bar{N}\gg 1, \tilde{t}\gg 1}{\sim} \Gamma(1+\gamma)\tilde{t}^{-\gamma}, \qquad \text{where} \qquad \gamma = -\frac{1}{\bar{N}\ln p_{\rm off}} > 0 \tag{S32}$$

and where Γ denotes the gamma function. Equation (S32) implies the power law presented in Eq. (8) of the main text, and its accuracy at long times is confirmed by the plots of Fig. (S7). As discussed in the main text in relation to trap models, here an exponential distribution of N combined with an exponential dependence of the relaxation time on N [Eq. (S29)] result in a power law distribution of the relaxation times. This distribution is apparent in the integral on the left of Eq. (S32), and eventually results in the power law relaxation. Note that the approximation of Eq. (S30) leads us to ignore a possible logarithmic dependence of $\sigma(t) \times t^\gamma$ on t, hence the small mismatch between the curves of Fig. S7.

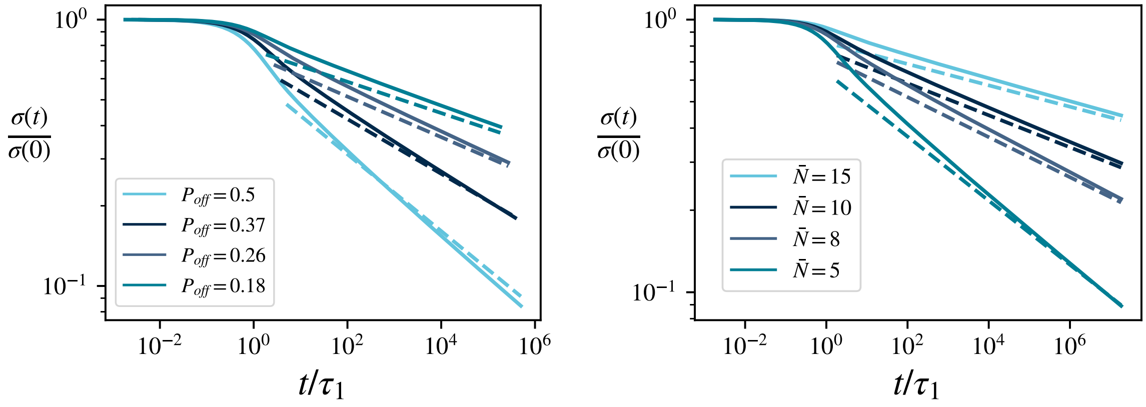

FIG. S7. **Comparison between exact and approximate expression of relaxation modulus**. The exact expression is given in Eq. (S29) (solid lines) and the approximate expression in Eq. (S32) (dashed lines).

S9. SCALING REGIMES FOR THE COMPLEX MODULUS

Here we derive Eq. (9) of the main text, the associated prefactors and its extension to $\gamma \geqslant 1$. In the linear response regime, the Fourier transform of the stress is related to that of the strain ϵ through the material's complex modulus G:

$$\sigma(\omega) = G(\omega)\epsilon(\omega). \tag{S33}$$

Denoting the Heaviside step function by H, we consider the response to a step strain $\epsilon(t) = \epsilon_0 H(t)$ and thus obtain $\sigma(\omega)$ by Fourier transforming Eq. (S29). Equation (S33) then yields

$$\int_0^{+\infty} e^{-i\omega t}\sigma(0)\sum_{N=1}^{N_{\rm sat}}\frac{p(N)}{1-p(0)}e^{-t/\tau_N}\,\mathrm{d}t = G(\omega)\int_{-\infty}^{+\infty} e^{-i\omega t}\epsilon_0 H(t)\,\mathrm{d}t \tag{S34}$$

where the bounds of the left-hand-side integral stem from the implicit assumption that $\sigma(t<0)=0$ in Eq. (S29). We compute both integrals in Eq. (S34) to find

$$\tilde{G}(\omega) = \sum_{N=1}^{N_{\rm sat}}\frac{p(N)}{1-p(0)}\frac{i\omega\tau_N}{1+i\omega\tau_N}, \tag{S35}$$

where $\tilde{G}$ is the dimensionless modulus obtained by normalizing G by the high-frequency elastic plateau $\sigma(0)/\epsilon_0$.

In the following we consider a generalization of Eq. (S28) where $p(N) \propto \exp(-N/\bar{N})$ for $N \leq N_{\rm sat}$ and $p(N) = 0$ for $N > N_{\rm sat}$. We analyze the scaling behavior of the storage modulus $G'(\omega) = \Re[G(\omega)]$ and the loss modulus $G''(\omega) = \Im[G(\omega)]$ computed from Eq. (S35).

In the high-frequency regime $\omega \gg \tau_1^{-1}$, the system displays a Maxwell-like rheology:

$$\tilde{G}'(\omega) \underset{\tau_1^{-1}\ll\omega}{\sim} 1 \tag{S36a}$$

$$\tilde{G}''(\omega) \underset{\tau_1^{-1}\ll\omega}{\sim} \frac{e^{-1/\gamma\bar{N}}(1-e^{-1/\bar{N}})}{[1-e^{-(1+\gamma^{-1})/\bar{N}}]^2}\frac{1}{\omega\tau_1}. \tag{S36b}$$

We now consider the intermediate frequency regime $\tau_{N_{\rm sat}}^{-1} \ll \omega \ll \tau_1^{-1}$ in the case $N_{\rm sat} \gg 1$. Provided we also assume $1 \ll \bar{N} \ll N_{\rm sat}$, the approximate power law response of Eq. (S32) applies and we obtain

$$\tilde{G}'(\omega) \underset{\tau_{N_{\rm sat}}^{-1}\ll\omega\ll\tau_1^{-1}}{\sim} \begin{cases} \frac{\pi\gamma/2}{\sin(\pi\gamma/2)}e^{-1/\bar{N}}\left(\frac{\omega\tau_1}{\bar{N}}\right)^{\gamma} & \text{if } \gamma < 2 \\ \frac{\gamma}{\gamma-2}e^{-2/\gamma\bar{N}}\left(\frac{\omega\tau_1}{\bar{N}}\right)^{2} & \text{if } \gamma > 2 \end{cases} \tag{S37a}$$

$$\tilde{G}''(\omega) \underset{\tau_{N_{\rm sat}}^{-1}\ll\omega\ll\tau_1^{-1}}{\sim} \begin{cases} \frac{\pi\gamma/2}{\cos(\pi\gamma/2)}e^{-1/\bar{N}}\left(\frac{\omega\tau_1}{\bar{N}}\right)^{\gamma} & \text{if } \gamma < 1 \\ \frac{\gamma}{\gamma-1}e^{-1/\gamma\bar{N}}\left(\frac{\omega\tau_1}{\bar{N}}\right) & \text{if } \gamma > 1 \end{cases}. \tag{S37b}$$

Finally, at low frequencies $\omega \ll \tau_{N_{\rm sat}}^{-1}$, the system again goes to a Maxwell-like rheology:

$$\tilde{G}'(\omega) \underset{\omega \ll \tau_{N_{\rm sat}}^{-1}}{\sim} A(\gamma, \bar{N})(\omega\tau_1)^2 \tag{S38a}$$

$$\tilde{G}''(\omega) \underset{\omega \ll \tau_{N_{\rm sat}}^{-1}}{\sim} B(\gamma, \bar{N})(\omega\tau_1), \tag{S38b}$$

where the functions A and B take simple forms in the $N_{\rm sat} \gg \bar{N}$ limit:

$$A(\gamma, \bar{N}) = \begin{cases} (1 - e^{1/\bar{N}}) \exp\left[-\frac{N_{\rm sat}(\gamma-2)+\gamma+2}{\gamma\bar{N}}\right] \Phi(e^{(2/\gamma-1)/\bar{N}}, 2, -N_{\rm sat}) & \text{if } \gamma < 2 \\ \left(1 - e^{-1/\bar{N}}\right) e^{(1-2/\gamma)/\bar{N}} \mathrm{Li}_2\left[e^{(2/\gamma-1)/\bar{N}}\right] & \text{if } \gamma > 2 \end{cases} \tag{S39a}$$

$$B(\gamma, \bar{N}) = \begin{cases} \frac{1-e^{-1/\bar{N}}}{N_{\rm sat}} \frac{\exp\left[(1/\gamma-1)N_{\rm sat}/\bar{N}\right]}{\exp\left[(1/\gamma-1)/\bar{N}\right]-1} & \text{if } \gamma < 1 \\ \left(1 - e^{-1/\bar{N}}\right) e^{(1-1/\gamma)/\bar{N}} \ln\left[\frac{1}{1-\exp\left[(1/\gamma-1)/\bar{N}\right]}\right] & \text{if } \gamma > 1 \end{cases}. \tag{S39b}$$

Here Φ denotes the Lerch zeta function defined as $\Phi(z, s, \alpha) = \sum_{n=0}^{\infty} z^n/(n+\alpha)^s$, which simplifies for $z \ll 1$ and $\alpha \gg 1$ (i.e. $p_{\rm off} \ll e^{-2}$ and $N_{\rm sat} \gg 1$) into $\Phi(z, s, \alpha) \sim \alpha^{-s}/(1-z)$. Li_2 denotes the polylogarithm function of order 2, which is defined as $\mathrm{Li}_2(x) = \sum_{k=1}^{\infty} x^k/k^2$. The three successive regimes described by Eqs. (S36-S38) are shown in Fig. S8.

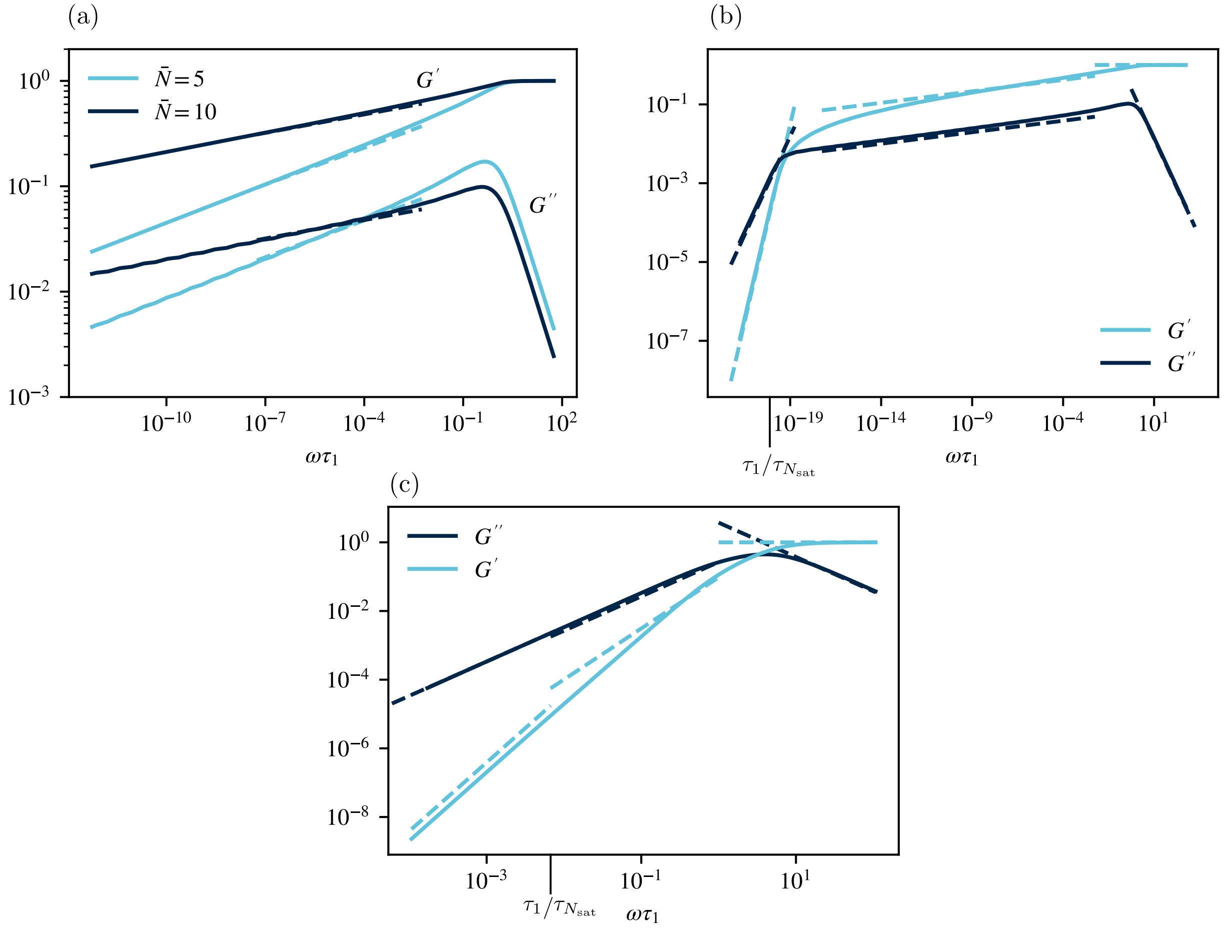

FIG. S8. Comparison between the storage and loss moduli computed from the exact expression Eq. (S35) (solid lines) and the asymptotic expressions of Eqs. (S36-S38) (dashed lines). (a) Plots in the large N_{sat} limit (here $N_{\mathrm{sat}} = 100$), showing a good agreement with the power law regime of Eq. (S37) for two values of $\bar{N}$ and for constant $p_{\mathrm{off}} = 0.18$ corresponding to $\gamma \simeq 0.116$ and $\gamma \simeq 0.0583$. (b) Plots for a smaller value of N_{sat} ($N_{\mathrm{sat}} = 30$) showing the three distinct asymptotic regimes. Here $\bar{N} = 10$ and $p_{\mathrm{off}} = 0.18 \Rightarrow \gamma \simeq 0.0583$. (c) Plot of the three distinct asymptotic regimes for a higher value of γ ($\bar{N} = 10$ and $p_{\mathrm{off}} = 0.935 \Rightarrow \gamma = 1.49$). The marker at $\omega\tau_1 = \tau_1/\tau_{N_{\mathrm{sat}}}$ denotes the expected position of the low-frequency crossover, while the high-frequency crossover is expected for $\omega\tau_1 \approx 1$.
